# Conducting clinical trials in persons with Down syndrome: summary from the NIH INCLUDE Down syndrome clinical trials readiness working group

**DOI:** 10.1186/s11689-022-09435-z

**Published:** 2022-03-23

**Authors:** Nicole T. Baumer, Mara L. Becker, George T. Capone, Kathleen Egan, Juan Fortea, Benjamin L. Handen, Elizabeth Head, James E. Hendrix, Ruth Y. Litovsky, Andre Strydom, Ignacio E. Tapia, Michael S. Rafii

**Affiliations:** 1 Department of Neurology, Division of Developmental Medicine, Department of Pediatrics, Boston Children’s Hospital, Harvard Medical School, Boston, USA; 2 Department of Pediatrics, Duke University School of Medicine, Durham, USA; 3 Department of Pediatrics, Kennedy Krieger Institute, The Johns Hopkins School of Medicine, Baltimore, USA; 4 Parent/Advocate, Virginia, USA; 5 Sant Pau Memory Unit, Department of Neurology, Hospital de la Santa Creu i Sant Pau, Biomedical Research Institute Sant Pau, Universitat Autònoma de Barcelona, Barcelona, Spain; 6 CIBERNED, Madrid, Spain; 7 Barcelona Down Medical Center, Fundació Catalana de Síndrome de Down, Barcelona, Spain; 8 Department of Psychiatry, University of Pittsburgh, Pittsburgh, USA; 9 Department of Pathology & Laboratory Medicine, University of California, Irvine, USA; 10 LuMind IDSC Foundation, Burlington, USA; 11 Waisman Center, University of Wisconsin, Madison, USA; 12 Department of Communication Sciences and Disorders, University of Wisconsin, Madison, USA; 13 Department of Forensic and Neurodevelopmental Sciences, Institute of Psychiatry, Psychology and Neuroscience, London, UK; 14 King’s College London & South London and the Maudsley NHS Trust, London, UK; 15 Division of Pulmonary and Sleep Medicine, Children’s Hospital of Philadelphia Perelman School of Medicine, University of Pennsylvania, Philadelphia, USA; 16 Alzheimer’s Therapeutic Research Institute, Keck School of Medicine, University of Southern California, Los Angeles, USA

**Keywords:** Down syndrome, Intellectual disability, Clinical trials, Clinical research, Research engagement, Recruitment

## Abstract

The recent National Institute of Health (NIH) INCLUDE (INvestigation of Co-occurring conditions across the Lifespan to Understand Down syndromE) initiative has bolstered capacity for the current increase in clinical trials involving individuals with Down syndrome (DS). This new NIH funding mechanism offers new opportunities to expand and develop novel approaches in engaging and effectively enrolling a broader representation of clinical trials participants addressing current medical issues faced by individuals with DS. To address this opportunity, the NIH assembled leading clinicians, scientists, and representatives of advocacy groups to review existing methods and to identify those areas where new approaches are needed to engage and prepare DS populations for participation in clinical trial research. This paper summarizes the results of the Clinical Trial Readiness Working Group that was part of the INCLUDE Project Workshop: Planning a Virtual Down Syndrome Cohort Across the Lifespan Workshop held virtually September 23 and 24, 2019.

## Background/introduction

The INCLUDE (INvestigation of Co-occurring conditions across the Lifespan to Understand Down syndromE) project was launched in June 2018 in support of a Congressional directive in the fiscal year (FY) 2018 Omnibus Appropriations. The directive called for a new trans-NIH research initiative on critical health and quality-of-life needs for individuals with Down syndrome (DS) and resulted from advocacy from the community, NIH commitment, and generous support from Congress. This program is developing a portfolio of scientific opportunities that span the spectrum from basic science to clinical research. Important insights are being gained from INCLUDE studies, and the capacity building (especially training for a cadre of investigators who will advance the field) that the program has accelerated provides confidence that this research trajectory will enhance the lives of people with DS.

The NIH INCLUDE project hosted a workshop titled “Planning a Virtual Down Syndrome Cohort Across the Lifespan Workshop.” Groups interacted via teleconferences and email during a 3-month preparation period. A 2-day face-to-face meeting was held virtually, September 23 and 24, 2019, at which all working groups presented their findings and participated in additional discussion and refinement of the initial work. At the workshop, specialists, including clinicians, researchers, advocates (parents and individuals with DS), as well as data scientists and biostatisticians, were brought together to discuss the creation of a virtual DS cohort across the lifespan. A final overview of the status of best practices for engaging and conducting clinical trials in the DS population was developed by the Clinical Trial Readiness Working group and is presented in the following sections. The focus of this paper is to outline current challenges and opportunities in clinical trial research in people with DS and to present recommendations for future work in this area.

### Current state of clinical trials in the Down syndrome population

Ongoing clinical trials with individuals with DS address a wide range of conditions and issues, such as Alzheimer’s disease (AD) dementia, cardiac disease, metabolic disorders and obesity, autoimmune disorders, obstructive sleep apnea (OSA), leukemia, behavioral, and mental health issues. Advances in medical care have improved overall health and extended the life span of individuals with DS. Current research is focused on development, cognition, with the goal of maximizing functional outcomes and improving their quality of life.

Great strides have been made in observational and behavioral research in DS, with thorough characterization of development and cognition [1, 2], as well as development of new tools to detect AD-related cognitive decline in adulthood [3, 4]. Working groups have also evaluated potential outcome measures that can be used in clinical trials in the areas of cognition and behavior [5]. However, despite these advances, clinical trial research, particularly that focused on cognition, has been slow to progress and poses unique challenges [6, 7]. Several issues have impacted the success of clinical trials in DS, and more broadly within neurodevelopmental disabilities (NDDs). These include barriers that individuals with DS and their families experience, ethical and logistical barriers that researchers face, and challenges with clinical trial research design and interpretation.

### Challenges and barriers for conducting clinical trials

The number of families and individuals with DS who participate in research and clinical trials is not sufficient to obtain valid and reliable results. NIH has created a DS Registry and while the numbers have increased over the past years, there is still a need for greater involvement of a larger number of participants.

#### Barriers faced by families

The biggest barrier is trust in most cases and/or the lack of exposure to the benefits of research. This results from limited community awareness and interest and therefore involvement in research, particularly in racially and ethnically diverse populations. Those who face disadvantage, be it due to factors such as disability, socioeconomic status, and/or ethnic/minority background, have expressed that it can be difficult for them to take part in research activities, and report their views and experiences are less often heard and addressed [8].

The logistical burden of participating in clinical trials is also cited as a challenge for families of individuals with DS, with families concerned about scheduling burden, family availability, overall time commitment, travel distance, out-of-pocket expenses, and frequency of visits [9]. These challenges are likely exacerbated in older individuals for whom caregivers are also older, or for individuals who are living in settings outside of the family home. Exploration of parent attitudes reveals that while many parents of individuals with DS generally support pharmacological trials, there are concerns about safety and long-term implications, potentially limiting participation [9, 10]. Decisions regarding participation in clinical trials may be dependent on the intervention target, such as medical versus developmental conditions/cognition or high priority symptoms such as communication or behavior [9]. People with DS may also have difficulty complying with study demands due to motor limitations, impulsivity, and limited attention span, and may require specialized testing administration, and customization.

#### Ethical and logistical barriers faced by researchers

Challenges with recruitment, retention, consenting/assenting, and logistics, safety, and efficacy have also long impacted clinical trial research in DS and special considerations are needed for success [6, 7]. Given the historical context of exploitation, there are issues surrounding the ethical and legal implications for conducting research with individuals with intellectual disability (ID) [11, 12]. For example, some researchers may be reluctant to include people with ID in their research for several reasons: they may inappropriately presume inability; they may be concerned about the capacity of the individual to understand risks and benefits and provide consent or assent to participate; or they may consider those with ID to be vulnerable and in need of protection from potential harms of research. While DS researchers may be well trained and experienced, and have comfort in addressing these issues, researchers less familiar with DS or ID, but who study health issues relevant to DS may not be. Choice of participants can also be problematic, as those with more severe impairments who may not be able to comply with study demands may not be well represented by findings originating from study participants with milder impairments who are better able to participate in research.

#### Challenges specific to clinical trials targeting cognition

There are specific challenges to clinical trials that target cognition, neurodevelopment, and neurodegeneration. These include heterogeneity within the population, interindividual variability, lack of standard endpoints to assess efficacy, placebo effects, reliance on informant-based questionnaires that require the same informant at multiple time points, and complexity in interpretation of findings. Current research most commonly involves those who have mild-moderate ID, and findings may not be generalizable to those with more severe impairment.

Clinical trials in DS have faced similar challenges to clinical trials in other NDDs. For example, in Fragile X, despite great success in understanding genetic and mechanistic causes of cognitive impairments and positive findings in mouse models, human studies of targeted pharmacologic treatments for cognition have not yet shown significant improvement in outcomes, and some pharmaceutical companies have discontinued further drug development [13]. However, there are many complexities to interpreting the available findings. It may be that studies are too quick to conclude that negative findings in a trial prove that a treatment is ineffective under all conditions or that the presumed underlying pathophysiological mechanisms are not valid [14]. For example, some treatments may be effective in certain subgroups of the population, at a different time in development, or under different conditions.

Without potential biomarkers of treatment response, human trials need to rely on behavioral outcomes, which reflect combined effects of many different factors such as learning and environment. Additionally, the behavioral outcome measures may not be sensitive enough to treatment effects, or best suited to the population studied or what is most relevant to families and people with DS. Standardized behavioral-based assessments have floor and ceiling effects and direct measurement testing batteries may over- or underestimate participant’s skills because they are not geared to their specific profile. Reliance on informant-based questionnaires is also particularly susceptible to strong placebo effects in NDDs [15].

Variability in cognition, behavior, language, and adaptive skills is seen in people with DS, and skills in these domains evolve across the lifespan, with environmental influences playing a role in ways that are not well characterized or understood. Furthermore, how medical and mental health conditions that occur at different times over the lifespan further influence neurodevelopment and outcomes.

While a longitudinal lifespan approach would help to answer important clinical questions that address both neurodevelopmental and neurodegenerative aspects of DS, there are many challenges to a lifetime approach as brain development, organization, maturation, and functioning evolve over time, and there may be critical windows for potential treatments to have desired effects. Additionally, pharmacological treatments targeting cognition or learning may need to be paired with structured therapy/teaching paradigms and study impact over longer time periods to adequately assess effectiveness of a drug that has the potential to enhance the learning process.

### Clinical trials readiness working group objectives

The Clinical Trial Readiness Working Group was provided with key questions and tasked with developing responses that could serve as a roadmap for efforts going forward in advancing clinical research efforts in DS and increasing participation among persons with DS in future clinical trials. The questions focused on (1) identification of approaches to facilitate recruitment and retention of research participants, including underrepresented groups, (2) cohort preparation for clinical trials with special considerations for clinical trials across the lifespan, and (3) building a pipeline of investigators with DS clinical trial experience. In exploring these questions, several themes emerged and fostered development of the recommendations that follow.

**Table:** 

**Key questions:** 1. Recruitment and retention: what approaches can facilitate recruitment and retention of families or participants to build a DS cohort and understand natural history, including underrepresented groups? 2. Cohort development across the lifespan: how can we ensure that cohorts are prepared for those with DS across the lifespan for future clinical trials? 3. Building an investigator pipeline: how can we build the pipeline of investigators who have DS clinical trial experience?	

### Recruitment and retention: strategies to promote engagement and inclusion

#### Engagement with individuals with DS, their families/care partners, and community advocacy organizations

Ensuring that research teams include members from the groups studied has been increasingly recognized as an important aspect of research involving individuals with disabilities [7, 15]. The shift from “researching on” to “researching with” has helped investigators understand the types of outcomes that are perceived to be useful within the community and can contribute valuable information to study design and implementation [16].

The Working Group identified areas of need and suggestions for recruitment and retention of research participants, with particular focus on engaging and involving groups often underrepresented in medical research. Involving individuals with DS and their families at all phases of research and fostering and maintaining research collaborations are necessary to promote engagement and advance clinical research while helping to further the understanding of needs and priorities.

Surveys, focus groups, and consultation and advisory feedback from families and participants can be particularly helpful when developing study questions and designing clinical trials. This can help to ensure understandability and feasibility of study assessments, outcome measures, information and consent materials, and will help researchers understand the relevance of the study question to participants and how they would like information conveyed. Some family members may have professional expertise that aligns with clinical and scientific goals. Investigators should take a community-engaged/community-based participatory research approach (CBPR), in which they collaborate with the community, and incorporate stakeholders’ expertise, values, and priorities in all steps of the research process [17].

Community engagement and outreach events may enhance outreach and education and can be conducted in person or virtually. Social media, websites, and newsletters from advocacy organizations can be used to advertise studies. Partnering with DS associations and societies (e.g., National Down Syndrome Society (NDSS), Global Down Syndrome Foundation (GDSF), LuMind Foundation, National Down Syndrome Congress (NDSC), Trisomy 21 Research Society (T21RS)), and religious and community groups, government, and advocacy groups will also support this effort. Importantly, an individual and family’s experience in a trial not only impacts retention for that trial but impacts recruitment for future trials. Partnerships can be further strengthened and promoted by engagement in which materials and information about ongoing studies can be disseminated. These efforts can help to promote partnerships, buy-in, and willingness for individuals to be contacted in the future for research opportunities.

#### Collaboration between researchers, clinicians, stakeholder groups, and advocacy organizations

In addition to strong community engagement, successful recruitment will rely on collaboration among clinicians and researchers, stakeholder groups, and advocacy organizations. Strong relationships with referring clinical programs, and in collaboration with clinicians who have pre-existing relationships with potential participants, will enable research to be considered as an extension of clinical care, with both clinical and research cultures existing synergistically. It is necessary to continue to build a strong network of clinical care centers for people with DS, and for researchers to collaborate closely with clinicians and to collaborate in study recruitment and study conduct, such as for collecting data from the clinic sample. Researchers should engage clinicians as partners and respect their involvement (e.g., the face of the study, collaborators on publications).

Partnerships between academic researchers and industry are needed to conduct large scale randomized, placebo-controlled clinical trials. Collaborations with researchers who focus on other areas of developmental disabilities may be useful to broaden the scope of endpoints. Continued collaborations with NIH and the Down Syndrome Medical Interest Group (DSMIG-USA), collaborations with researchers and clinicians, basic scientists, and clinical researchers are needed to further this effort.

#### Engagement of underrepresented groups

Engagement and involvement of minority groups who are underrepresented in biomedical research is a particular challenge. It is necessary to incorporate strategies that address cultural and language barriers, such as adapting information, providing translations for written documents and interpreters for oral communications, and targeting recruitment. It will be necessary to identify strategies to gain information about the ages, ethnicities, and races of people with DS in research catchment areas and clinical research sites and to work closely with the community to engage participants. Recruiting people from various backgrounds and minority groups can also be enhanced by inclusion of people on the research team that captures this diversity.

#### Providing accessible information

A careful and thorough process of communication is needed when conducting research in individuals with DS. Use of accessible information and repeated opportunities to communicate information and check for understanding are needed. Novel ways to address informed consent, such as through picture-based consents and electronic consenting platforms (eConsenting), are needed. For example, the U.S. Food and Drug Administration has guidance on use of electronic consents [18]. Involvement of individuals with DS and their families in reviewing consent materials is especially important. Individuals with DS may be inclined to give socially desired answers, so checking for understanding and use of visually accessible information is needed.

Timely and accurate dissemination of important findings, including safety information and results, is imperative. New and creative ways to acknowledge participation in research and to share and distribute study results are crucial. Information should be shared in formats that are appropriate to the audience, such as through a newsletter or infographic, rather than a journal article. For example, T21 RS COVID-19 infographic summaries, vetted through stakeholders and advocacy groups, have proved to be a successful method for dissemination of key research findings [19]. Providing a platform for patients and research participants to share their experience in research with others may also be a valuable tool for all stakeholders.

#### Reducing study burden

Trial burden may be decreased by using telemedicine and home visits, or by coordinating research with clinic visits or on nights or weekends, so as not to interfere with school, work and other activities and appointments. Budgets should reflect the additional cost required to bring in research staff on off-hours. It is also important to ensure that families, participants and care partners are appropriately supported to attend visits (e.g., travel arrangements and reimbursement, compensation for use of care hours, flexibility in timing of visits, adequate breaks during study visits.) Travel for adult participants, especially, can be a barrier to participation, and travel expenses should be included in the budget. Group travel for individuals with Down syndrome living together in a group home can save money and be enriching for participants. In addition, remote visits, such as via telemedicine, could also reduce travel burden for participants and expand research catchment areas. Direct to patient trials in which study treatments and procedures are delivered in the participant’s home could also be considered to optimize recruitment and minimize burden to patients and families [19].

Data collection in research areas of high interest (e.g., sleep, behavior, cognition, mental health) are extremely labor- and time-intensive to conduct. Development of select web-based, interactive assessment tools (point-click-scroll) will be helpful to partially replace the need for in-person visits. Some studies are also completed entirely online, such as the Alzheimer’s Prevention Trials web-based research study [20]. Efforts are needed to begin to validate these tools and compare to gold-standard assessments, and also to collect data longitudinally to determine test-retest reliability. Feasibility trials/pilot studies are likely to be needed before larger scale trials are conducted.

#### Legal and ethical considerations

Autonomy and self-determination must be respected and balanced with the responsibility to protect vulnerable individuals from potential risks of participation in research studies, thus the informed consent process, in particular, demands careful planning.

Given the ID associated with DS, as well as potential emotional/behavioral conditions that influence decisions, individuals with DS must be supported in research decision-making. In many cases, and depending on the legal framework, a surrogate or proxy decision maker is involved, and a legally authorized representative makes decisions about participation. Many individuals are comfortable receiving assistance from trusted family and friends to help them with decisions. Even for individuals who cannot provide legal consent, they may be able to express their thoughts and feelings about participating and exercise some choice. Assent—or willingness of the prospective participant to go along with or not object to the study—is essential, even when there is a surrogate decision-maker.

Special consideration is also needed for recruitment/consenting of aging adults with DS or adults living in group homes who may lack a legally authorized representative or family informant. The legally authorized representatives of adults with DS often change over time, for example, from an aging parent to a sibling or professional caregiver. Each state or country has different regulations around consent for individuals with disabilities. This must be determined in advance to inform clinical trial development, with particular consideration of multisite/multiregional studies, and local/state regulations must be taken into consideration.

### Cohort development across the lifespan

Cohort development across the lifespan is critical for readiness for future clinical trials. These trial ready cohorts might accelerate research for several clinical needs (e.g., AD and OSA), thus reducing the costs of performing different clinical interventions. Further development of reliable and valid clinical outcome measures across the lifespan and across a wide range of developmental abilities are needed. Longitudinal studies are needed to better understand the natural history of DS and its associated conditions, to identify DS-related norms, to help identify subgroups with characteristics, to understand what childhood factors may predispose to risk or resilience, and who may be good candidates for preventative interventions.

Many medical issues for adults with DS such as obesity and sleep apnea begin in childhood and childhood antecedents may play an important role in how these, and other adult-onset conditions, evolve. For example, AD, which is one of the most prevalent and challenging conditions for adults with DS, has genetic but also likely environmental origins early in childhood [21]. Some research in the general population has shown associations between AD and increased exposure to adverse childhood experiences (including living in custody and difficult experiences with teachers) [22, 23]. In addition to research trials in older adults, longitudinal studies in DS will be needed in DS to understand early risk factors and the potential impact of childhood experiences. Additionally, the association between OSA in adults with AD is currently under investigation in the general population [24]. With high rates of OSA in people of all ages with DS, it will be important to understand whether OSA in childhood might impact later risk of AD. Similarly, research in typically developing children has shown that persistent sleep problems through childhood are associated with worse behavioral outcomes and quality of life. A longitudinal study in individuals with DS might be able to provide important insight into the association between sleep and health outcomes across the lifespan [25]. Furthermore, there are conditions that arise specifically in childhood, for example DS-associated arthritis that can affect long-term physical outcomes. DS-associated arthritis is under-recognized, has a more severe phenotype, and is difficult to treat. It can result in disruptions in motor development, unpredictable medication toxicity and significant long-term physical disability [26].

Ongoing work to accelerate the development, evaluation, and validation of new trial-enabling standardized outcome measures for use over the lifespan is also needed. Many measures currently used in neurotypical children and adults cannot be used in those with DS. Cognitive measures are needed that are normed for individuals with DS and age appropriate, which are sensitive to bidirectional change (i.e., can detect developmental growth and skill acquisition as well as decline), and that can be used in individuals with a wide range of abilities, including those with more severe ID [5].

While longitudinal lifespan studies are logistically challenging, through close collaboration among the DS-community and advocacy organizations, specialized pediatric and adult clinical DS programs, and researchers, large registries and clinical databases of well-characterized cohorts can be created and shared. NIH-funded Data Coordinating Centers [27] can lead these efforts by serving as a platform for linking investigators for collaborative research. Additionally, with opportunities for clinical research throughout the lifespan, more touchpoints and ongoing engagement can be fostered for future recruitment into clinical trials. Exposure to research results and sharing the successes in a case study can help build trust. Also, most publications are usually scientific and do not reach the target population. The recommendation is to publish for the target population on their websites and share these in DS conferences to convey results and findings to the population.

The timing of interventions will be critical to consider. In younger children, developmental change will be expected in the absence of intervention, and in older individuals, there may be points at which aberrant neurodevelopment or the later neurodegenerative cascade, are too far underway such that neurobiological consequences are irreversible and functioning cannot be altered. Additionally, specific brain areas impacted in DS mature at different rates and manifestations of their disrupted development change across the lifespan, suggesting that some pharmacological targets may be most effective at different time points in development. For these reasons, targeting younger individuals for treatment and prevention trials, in addition to older adults, may be an additional important approach.

Clarity is needed on a regulatory pathway for new drugs for DS, as the typical progression of drug development that is initiated in neurotypical adults, then children, may be problematic. It is not clear that demonstration of safety in a neurotypical population would predict safety in DS. In addition, individuals with DS are at increased risk for numerous co-occurring conditions that require chronic medications and very little is understood about drug biotransformation in DS, despite observed differences in drug response and toxicity [28–30]. Dedicated pharmacodynamic and pharmacokinetic studies, such as those conducted through the Pediatrics Trials Network—currently investigating metabolism of Guanfacine in Down syndrome—are needed more broadly in DS to determine drug disposition and response relationships, and to establish safety and dosing recommendations across the lifespan. Additionally, training and expertise are needed for identifying and managing potential adverse events, particularly when participant reporting of side effects and problems may not be straightforward.

Coordinated efforts across many sites will be needed to build large cohorts, and leverage existing infrastructure, including Alzheimer's Disease Research Centers (ADRCs), Centers for Excellence in Developmental Disabilities (CEDDs), Intellectual and Developmental Disability Research Centers (IDDRCs) and others. Harmonization of test batteries across studies and across age groups is desired so that results can be compared and aggregated across studies. Several strategies can help with cohort development, including continued support of DSConnect and similar efforts to build a registry of interested participants, continued work to prepare and maintain lists and coordination of existing cohorts through the Data Coordinating Centers, and ensuring that new research study design is tailored and compatible with existing cohorts. This involves ongoing work to determine a minimum set of core data elements and informative measures, and to create standard operating procedures to facilitate systematic collection of biological and standardized and consistent behavioral data across studies and sites.

### Building an investigator pipeline in Down syndrome research

Coincident with bolstering clinical trial participation by members of the DS community, there is a critical need to have clinical research sites trained and resourced to conduct such studies. The Working Group offered several recommendations to support this effort. The INCLUDE infrastructure can be used to bring in early stage as well as established investigators into the DS research space by capitalizing on existing training mechanisms, such as NIH Career Development and Training pathways (e.g., K23, T32 training awards).

Interest in DS research can be expanded and fostered by engaging clinicians, researchers, and trainees including those who are not focused solely or primarily on DS or even ID and providing training and mentorship. To encourage DS clinicians to participate, it will be necessary to offer training bootcamps to provide skills in clinical trial design, implementation, and funding, as well as ongoing mentoring opportunities.

In order to encourage other researchers to include people with DS or other intellectual and developmental disabilities in their research, it will be necessary to provide training on how to interact with and include people with DS.

Training opportunities can be built into NIH clinical trials infrastructure such as the Pediatric Trials Network (PTN) and Alzheimer’s Clinical Trials Consortium (ACTC). For example, ACTC launched the IMPACT AD training course in 2020. Lectures are given by national leaders in Alzheimer’s Disease and Related Dementias (ADRD) trials, and attendees have the opportunity to work closely with the field’s top investigators in small group workshops. Groups such as the ADRC can be encouraged to recruit and help train investigators interested in focusing on DS. There is now a DS module developed by the National Alzheimer’s Coordinating Center (NACC) that can be implemented at all ADRCs. It will be important to develop international links and collaborative networks via organizations such as the Alzheimer’s Association International Society to Advance Alzheimer’s Research and Treatment (ISTAART) and T21RS to capitalize on global expertise. All NIH funded ADRCs also now include a Research Education Component that could be coordinated to provide training modules on the importance of research in people with DS and Alzheimer disease.

The PTN also launched a PTN Down Syndrome 2020 Virtual meeting to introduce to the larger network and interested collaborators the therapeutic challenges faced by children with DS; opinions of community advocates, individuals, and parent/family on engaging in current and future research; lessons learned from current efforts to enroll patients with Down syndrome into an active PTN study; and input on a prospective randomize clinical trial protocol in development.

Existing IDDRCs can also be leveraged to fund postdocs and/or PhD positions in their labs to draw students from other fields into the DS space. This can be accomplished by offering CME activities and presenting workshops/symposia at national meetings focused on DS/ID [e.g., DSMIG-USA, DSMIG-UK, Child Neurology Society (CNS), American Academy of Neurology (AAN), AAIDD-Gatlinburg, and American Academy of Developmental Medicine and Dentistry (AADMD)] or with stakeholders not focused solely or primarily on DS or even IDD (e.g., AMA, APA, AAIC.) Finding ways to support student/young investigator attendance at these meetings will also be important.

## Discussion/key points

Advancing research in DS will require ambitious and collaborative efforts that reach across the lifespan and include researchers, clinicians, individuals with DS and their families, advocacy groups, and industry. The Clinical Trial Readiness Working Group has identified many challenges and opportunities for these efforts and has suggested approaches in developing some of the infrastructure and methods needed to conduct clinical trials successfully in people with DS.

Specifically, the Working Group highlighted the importance of the following:

1. Stakeholder engagement from the inception of any research project.
2. Ethical, accessible (culturally and cognitively appropriate) recruitment materials, ensure collection of assent as part of the consenting process if a legal guardian is providing consent, study materials, and dissemination of results available in different languages.
3. A lifespan approach from time of diagnosis (including prenatal diagnosis) to death both to evaluate childhood antecedents of adult disease or decline and to evaluate effects of interventions.
4. Development of appropriate tools to assess outcomes in health, quality of life, cognitive functioning and behavioral/mental health that can be used across institutions and age ranges.
5. Workforce development of a pipeline of researchers and clinicians to collaborate in ongoing research efforts.

## Conclusions

As we develop more advanced infrastructure to translate research towards clinical benefit for individuals with DS, it will be critical to continue the efforts described by the Working group to achieve the best possible outcomes and ensure that important research opportunities are accessibility for all.

## Data Availability

Not applicable.

## Abbreviations

NIH INCLUDE: National Institute of Health INvestigation of Co-occurring conditions across the Lifespan to Understand Down syndromE
DS: Down syndrome
NDD: Neurodevelopmental disabilities
ID: Intellectual disability
NDSS: National Down Syndrome Society
GDSF: Global Down Syndrome Foundation
NDSC: National Down Syndrome Congress
T21RS: Trisomy 21 Research Society
DSMIG-USA: Down Syndrome Medical Interest Group-USA
OSA: Obstructive Sleep Apnea
ADRC: Alzheimer's Disease Research Centers
CEDD: Centers for Excellence in Developmental Disabilities
IDDRC: Intellectual and Developmental Disability Research Centers
PTN: Pediatric Trials Network
ACTC: Alzheimer’s Clinical Trials Consortium
ADRD: Alzheimer’s Disease and Related Dementias
IMPACT-AD: Institute on Methods and Protocols for Advancement of Clinical Trials in ADRD
NACC: National Alzheimer’s Coordinating Center
ISTAART: International Society to Advance Alzheimer’s Research and Treatment
CNS: Child Neurology Society
AAN: American Academy of Neurology
AAIDD: American Association on Intellectual and Developmental Disabilities
AADMD: American Academy of Developmental Medicine and Dentistry
AMA: American Medical Association
APA: American Psychological Association
AAIC: Alzheimer’s Association International Conference
